# Adaptation and integration of the thinking healthy programme into pregnancy schools in Istanbul, Turkey

**DOI:** 10.1186/s12884-023-05572-y

**Published:** 2023-04-12

**Authors:** Perran Boran, Melike Dönmez, Najia Atif, Anum Nisar, Ezgi Barış, Mahmut Caner Us, Zeynep Meva Altaş, Seyhan Hıdıroğlu, Dilşad Save, Atif Rahman

**Affiliations:** 1 School of Medicine, Division of Social Pediatrics, Marmara University, Istanbul, Turkey; 2 School of Medicine, Department of Psychiatry, Marmara University, Istanbul, Turkey; 3 Human Development Research Foundation, Islamabad, Pakistan; 4 School of Public Health, Xi’an Jiaotong University, Xi’an, China; 5 Institute of Health Sciences, Social Pediatrics Doctorate Program, Marmara University, Istanbul, Turkey; 6 School of Medicine, Department of Public Health, Marmara University, Istanbul, Turkey; 7 Institute of Psychology, Health & Society, University of Liverpool, Liverpool, UK; 8 Institute of Population Health, University of Liverpool, Waterhouse Building, Block B, Brownlow Street, Liverpool, L69 3GF UK

**Keywords:** Perinatal depression, Maternal mental health, Psychosocial intervention, Thinking Healthy Programme, Qualitative analysis

## Abstract

**Background:**

Perinatal mental health is a major public health concern. In Turkey, public hospitals operate pregnancy schools which provides an opportunity to integrate an evidence-based Thinking Healthy Programme (THP) for perinatal depression. The aim of this study is to adapt the THP for universal use in the group setting and to understand its acceptability and feasibility for integration into the existing antenatal care programme for both face-to-face and online delivery.

**Methods:**

Following an expert-led adaptation process using the Bernal Framework, field testing was conducted on a group of women and facilitators followed by in-depth interviews (n:8) and group discussions (n = 13). Data were analysed using Thematic Framework Analysis.

**Results:**

Minor but significant adaptations were made to the individually delivered THP for use in the universal group pregnancy schools. Initial findings indicate that the THP-group version was acceptable to its target population and could be integrated into the antenatal care plan for delivery during face-to-face and online group classes.

**Conclusion:**

THP is transferable to the Turkish cultural and healthcare context. The THP–group version has the potential to add value to Turkey’s existing perinatal healthcare programme.

## Introduction

Globally, depression accounts for the second leading cause of disease burden in women of child-bearing age [1]. In Turkey, the prevalence rate of perinatal depression has been estimated between 20 and 40%, reflecting the global average of 25% [2, 3]. Untreated perinatal depression is of concern not only because of its effect on maternal health but also on mother-infant bonding, child care, and the long-term impact on the infant’s physical and cognitive development [4]. This highlights the need to develop or adapt evidence-based interventions to make them culturally and contextually relevant to the target population. The US Preventative Services Task Force found convincing evidence that counselling interventions are effective in preventing perinatal depression [5]. Similar findings have also been reported by a recently conducted meta-analysis of psychological and psychosocial interventions aimed at the prevention of perinatal depression and anxiety [6]. The commonly used effective ingredients in these interventions included psychoeducation, social support, cognitive-behavioural, interpersonal, and group therapies [6]. Furthermore, preventive interventions were found acceptable and feasible in a variety of settings. These include urban, rural, online, or multiple settings, with a universal or targeted focus such as on adolescent mothers, women in public assistance, or primiparous mothers and targeting either only postpartum depression/anxiety or focusing on both [6]. Interventions were delivered either by professionals with mental health background such as social workers, clinical psychologists, or mental health nurses, or by lay professionals including midwives, facilitators, and health visitors. For scaling up, such interventions need to be integrated into the routine maternal mental health (MH) programmes [7, 8]. In resource-constrained settings, delivering interventions in group settings is likely to be a feasible option. There is evidence that group interventions are less resource-intensive and are possibly a cost-effective way of improving accessibility [9]. Furthermore, there is evidence that group CBT is an effective psychological intervention in the treatment of perinatal depression [10].

In Turkey, antenatal care for women is prioritized with the support of the Turkish Ministry of Health. The public hospitals operate ‘antenatal pregnancy schools’, and all women attending antenatal care (usually between 12 and 35 weeks of gestation) are invited to attend 5 weekly group sessions that incorporate education about pregnancy and newborn care.

These groups provide an opportunity to integrate preventative interventions for perinatal depression into the antenatal care programme.

However, to our knowledge, no psychological or psychosocial intervention has been integrated into the existing antenatal care programme for the prevention of depression. One such intervention with the potential of integration is the cognitive behaviour therapy (CBT) based Thinking Healthy Programme (THP) [11]. It is an evidence-based psychosocial intervention recommended by the World Health Organization (WHO) as the first-line management of perinatal depression in primary and secondary care settings [12]. It has 16 home-based sessions covering the period from the third trimester of pregnancy to 10 months postnatal. It is delivered individually by community health workers (CHWs). The THP manual has already been translated into the Turkish language by a bilingual health expert (PB) and is available on the WHO website [13, 14].

Our study aims to (a) adapt the THP reference manual-Turkish version for use in the group setting as a preventative intervention, (b) understand the relevance and acceptability of the adapted version to its delivery agents and expectant women, (c) explore its feasibility for integration into the existing antenatal care programme and, (d) explore online delivery.

## Methods

### Procedures

#### Step 1: Adaptation of the THP reference manual-Turkish version for universal use in the group setting

The Bernal Framework for adaptation was used to adapt THP for universal use in the group setting [15]. To facilitate the process of adaptation an expert group was formed, which included the author of the original intervention manual (AR), a THP master trainer (NA), health experts (PB, MD), potential users (i.e. expectant women), and a delivery agent (i.e., pregnancy school nurse). The expert group considered the target population’s cultural, linguistic, and socioeconomic context. Cultural adaptation aimed to maintain the core elements of the intervention with systematic adjustments such as adding some cultural content to the surface structure (illustration materials with cultural clothes or foods, words, and expressions appropriate for the target population) and to the deep structure of the program (addressing cultural norms, beliefs, values and lifestyles, instructions with cultural correspondence) [16].

In order to incorporate the THP intervention into the routine pregnancy schools (offered to all expectant women) the expert group focused on the THP sessions given during the antenatal period. These sessions focused on psychoeducation, improving women’s well-being, mother-baby bonding, and enhancing their social support. The core strategies included; building an empathetic relationship with the women, behavioural activation, thought challenging, problem-solving, and family involvement. To incorporate the contents of THP antenatal individual sessions into the antenatal group classes adaptations were made (Table 1).

#### Step 2: Field-testing of the adapted THP

The purpose of our field testing was to deliver the THP-adapted sessions and to obtain feedback about their acceptability, comprehensibility, and cultural relevance from the expectant women who received the sessions and the nurses who will deliver the sessions [17].

The field testing was conducted at Marmara University Hospital in Istanbul, Turkey, which is an affiliated hospital with the Ministry of Health of Turkey and located within the catchment area of the First Region of the Istanbul Public Hospitals Union. The First Region covers socioeconomically deprived districts of Istanbul and a population of about 1.5 million. The population is characterized by low literacy (about 30% of women have not attended school), high rates of internal migration, low socioeconomic status, and poor maternal and child health indicators. The participants were purposively selected to represent a cross-section of the population. Eight expectant women were recruited after obtaining their informed consent. The participants’ age ranged from 24 to 31 years, and years of schooling between 8 and 17 years. The participants received the intervention as part of their routine antenatal classes. The intervention for field testing was delivered by the Master Trainers (MD and PB).

#### Step 3: Exploring the acceptability and feasibility of the adapted THP

In-depth interviews were conducted with the participants (n = 8) after they had received the THP-group version, in order to assess its acceptability, comprehensibility, and cultural relevance until data saturation was reached. The topic guide was developed and pilot tested before being finalised. Topics were elaborated based on previous cultural adaptation and feasibility studies of the original THP conducted in Pakistan and India [18]. The interviews were conducted by independent research members who were not involved in the adaptation and/or delivery of the intervention (SH,DS). The in-depth interviews were face-to-face and conducted in the hospital setting in private interview rooms. All interviews were audio recorded and lasted 30–45 min.

Ethical approval was obtained from the Marmara University School of Medicine Clinical Research Ethics Board (09.2018.389).

#### Step 4: Exploring the acceptability and feasibility of the online delivery of the adapted THP

Prior to the commencement of the feasibility study, Turkey was hit by the COVID-19 pandemic, and several precautionary measures were adopted on March 20, 2020. Consequently, face-to-face pregnancy classes were changed to remote self-help classes. In order to understand facilitators and barriers to delivering the online THP- group version, a series of group discussions were conducted. Participants invited to take part in these discussions included the facilitators (n:4) and expectant women (n:9), purposively selected, who were registered for the routine antenatal classes at Marmara University before they attended the usual classes. The discussions were conducted online via Zoom. The key points are listed below:

1. Online delivery of sessions requires the recipients to have a smartphone/laptop, internet access, and basic technology literacy.
2. Some smartphone owners might need internet packages to allow access to online intervention.
3. Lack of space at home can create a barrier to privacy needed to receive the session.
4. Families’ preference for the babies’ wellbeing can lead to a lack of family support for the women, and create barriers for them to receive the intervention.

Overall, the findings from the qualitative study indicated that the THP-group version, when delivered face-to-face, was found comprehensible, acceptable, relevant, and useful to most of the participants. However, based on the discussions about its online delivery, some further adaptations were made. Table 1 represents the list of final adaptations made for the delivery of THP-group sessions during the online antenatal group classes.

### Data analysis

Thematic framework analysis was used to analyse the data. Audio recordings were transcribed verbatim, and any personal identifying data were removed. Initial coding schemes were generated by two independent interviewers using a conventional content analysis approach. Similar codes were grouped to form sub-themes. The sub-themes, based on their similarities and differences, were clustered together to form themes. The process of development of the thematic framework was supervised by PB, and any inconsistencies were discussed with the research team to clarify the findings.

## Results

Analysis of the data generated four themes which are described below.

### Theme 1: Cultural relevance and usefulness of the adapted THP

The majority of the women found the intervention material comprehensible, relatable, and beneficial. They reported an increase in their knowledge and felt better equipped to provide care to their babies.

*“I would definitely want to be informed (about the perinatal period, stress, and feelings). My mother and grandmother have been through this period, but this is my first pregnancy. Yes, there are some books, but it is not possible to learn everything from books. Every day, we learned something new”.* (28-year-old, pregnant woman).

*“Although we talk about the mother-baby relationship in the lessons, pregnancy and postpartum period is very baby-centered in most of the families living nearby. Mother’s feelings are left in the background. I believe that this program can be very effective in showing the importance of maternal mental health in pregnancy schools”*. (23-year-old, nurse)

Problem-solving and stress management strategies were the most frequently reported beneficial techniques. They found problem-solving skills help to deal with their everyday issues and breathing exercises useful to manage their anxiety.

*“Problem-solving and deep breathing exercises were a huge help because these exercises taught me to cope better with anxiety and to think positively”.* (29-year-old pregnant woman)

Most women reported Mood Chart was helpful in monitoring their mood. It also helped husbands to recognize their wives’ feelings and offer better support to them. The Mood Chart in the original THP is used for encouraging mood monitoring and behaviour activation [12]. However, Health Charts such as Diet and Rest charts were not found to be very useful. These charts were for the women to follow their diet and daily activities by writing their daily intakes or recording their activities. This might be explained by the fact that women were already receiving such information from their pregnancy schools.

### Theme 2: Acceptability of the format of delivery

All the participants found the delivery of the programme in a group setting acceptable and valuable. They reported a positive experience of interacting with other participants and their facilitators and appreciated the friendly environment created in a group setting.

*“I felt very comfortable interacting with other women in the group and expressing my feelings. The group environment was very friendly, which made me comfortable and safe in expressing my feeling”.* (29-year-old pregnant woman)

The group format helped the women to realize that they were not alone in their fears and anxieties. They valued the emotional support offered by the group and felt safe sharing their problems without fear of being judged. They appreciated the suggestions offered to them.

*“My favourite thing was sharing and listening to each others’ problems and finding solutions”.* (26-year-old, pregnant woman).

*“In addition to the women’s physical health, I think it is very valuable to focus on the issues related to women’s mental health. Role plays, and sharing in-group experiences were very demonstrative and helpful for pregnant women. They normalize their worries about their pregnancy period with the help of these.”* (40-year-old, nurse).

While most participants were satisfied with the group, some wanted the sessions to be of shorter durations and more frequent during the week. A few participants also expressed their wish to have more sessions as they felt relaxed and suggested sessions to continue after the baby was born. One participant expressed her dissatisfaction with the timing of the session as it was falling during her lunch hour.

### Theme 3: Improved well-being and interpersonal relationships

The majority of the women reported applying the skills and knowledge learned during the sessions to improve their well-being. The breathing exercise was found particularly useful.

*“There were not only theoretical discussions. There was also practice work such as exercises and breathing exercises. I really felt relieved when I practiced them at home. I felt better and better every week”.* (28-year-old, pregnant woman).

Some women reported the intervention not only benefitting them but also their family members. One woman during her interview stated how it helped her and her husband to change their unhelpful thinking patterns.

*“The programme is helpful for my unborn baby, my husband, and my mother-in-law. I was able to change not just my thoughts into healthier ones, but also my husband’s”.* (28-year-old pregnant woman)

Many participants reported improved relationships with their husbands and in-laws. They attributed this to their improved confidence and better communication. They were able to share their concerns and ask for help. A woman whose marriage was on the brink of breaking stated;

*“I did not have good communication with my husband, and I was even thinking of getting a divorce, but it has changed. We are spending more time together, listening to each other, and having a more positive point of view”.* (31-year-old pregnant woman)

Not only was their relationship improved with their families, but it also helped them to bond with their unborn baby. During the interviews, they reported getting into the habit of talking and singing to their babies.

*“I found it difficult to bond with the baby for the first 4 to 5 months. But with the support of the programme I communicate more, make her listen to songs, and tell her stories, which makes me feel better”.* (30-year-old, pregnant woman).

### Theme 4: Appropriate delivery agent

The majority of the participants described their facilitators as genuine, supportive, and empathetic. They felt that the facilitators listened to them, made them feel valued, allowed them to ask questions, and responded to their queries adequately. The facilitators’ positive attitude made them feel comfortable and encouraged them to express their feelings.

*“The facilitator addressed each of us by our names and listened eagerly by spending her time. She engaged all of us in the problem-solving process, asking what would you do if you are in her shoes. The facilitator encouraged us to feel included and valued within the group”.* (31-year-old pregnant woman)

**Table 1:** Adaptation of the THP for incorporation in the routine online pregnancy schools

Overall adaptations for the THP – Group version		Rationale
Language	Translation of the manual into the Turkish language. The key messages were simplified and generalised to target all expectant women.	To make intervention universal instead of targeted for perinatal depression. To allow generating discussions in the group.
Context	The illustrations were re-draw to depict Turkish women in everyday scenarios	To make illustrations relevant to its target population
Concept: the core strategies	The facilitators used; (a) empathy to build rapport with the group members, (b) illustrations for psychoeducation and to highlight the importance of thought challenging, (c) mood charts for encouraging mood monitoring and behaviour activation, and (d) everyday examples for enhancing problem-solving skills.	Original THP core strategies were aimed to improve depression symptoms through the delivery of individual sessions. The THP-group version used these strategies for psychoeducation in group settings and to prevent depression.
Content:	The introductory session was split into sessions 1 and 2. Session 1: Engaging the participants and introducing the THP-group version. Session 2: Psychoeducation and problem-managing skills	Splitting the introductory session into 2 sessions will allow more time for: 1) rapport building, 2) group gelling, 3) introducing the programme, relaxation exercise, and introducing 5 steps of problem-solving skills, 4) raising awareness and generating discussions on the importance of emotional well-being during pregnancy
	Sessions 3: Personal Health Diet chart and rest charts are removed	These topics are addressed during the routine pregnancy schools and therefore not needed
	Session 4: Relationship with the baby More information is added about the importance of 1000 days, maternal responsiveness to the baby’s needs, breast feeding, and colic babies.	These topics are considered important by a co-author PB, who is a paediatrician by professional
	Session 5: Relationship with people around the mother The ending of session 5 focuses on concluding the group	This will allow the participants to give closure to the group and give feedback on their experiences
The organisation	Marmara University hospital under the Turkish Ministry of Health	Incorporating the THP-group sessions at ‘pregnancy schools’ at public hospitals
The individuals	Delivery agent: Nurses	Antenatal nurses to deliver THP- group sessions during the routine pregnancy schools to all expectant women
The format	Mode of delivery: On-line delivery of sessions	The COVID pandemic poses a risk for the face-to-face delivery of the sessions.
	Duration: 1 h except for the introductory session of 90 min with a break in between the routine program	Shorter duration and breaks to allow pregnant women to rest during sessions.
	Time of sessions’ delivery: Noon	Daily housework is already done and school-aged children were offline and to facilitate working women’s attendance
	Accessibility: Smartphone ownership and internet availability	Providing internet packages will allow the participants to receive online sessions

## Discussion

This paper describes the process for adaptation of the targeted perinatal depression intervention called the Thinking Healthy Programme (THP) for universal use in the group setting. The adapted version was designed to be integrated into routine pregnancy schools as part of their antenatal care plan. The adaptation of the THP required minor but significant changes. Initial findings indicate that the THP-group version was relevant and acceptable to its target population and could be integrated into the antenatal care plan for delivery during face-to-face and online group classes.

The original THP was created in Pakistan following extensive formative research and a rigorous evaluation via a randomised controlled trial [11, 19]. The global WHO version of the THP, which was produced by a team of experts including the original author, advocated local changes before deployment but did not give a defined approach for doing so [12]. Researchers have employed several overlapping frameworks for adaptation: In Peru, the Replicating Effective Programs (REP) framework was used to guide the THP’s implementation process [20]. The authors relied on a pre-translated Spanish version of the THP conducted by a community-based organisation for their local programmes a description of and rationale for the adaptations were not provided. In China and Vietnam, a more structured adaptation approach based on the WHO’s recommended International Management of Childhood Illnesses adaption toolkit was employed [21–23]. Our methods use elements of this toolkit but employ a more rapid but replicable methodology.

The use of CBT and CBT-based interventions for maternal mental health has been used widely and accepted as valid treatment tools [24, 25]. The THP-group version retained all the key CBT strategies used in the original THP, such as psychoeducation, problem-solving, social support, thought challenging, and behaviour activation. In the group version targeting all women rather than only those diagnosed with depression, these strategies were simplified and used mainly to raise awareness about improving well-being, strengthening mother-baby bonding, and improving interpersonal relations for the prevention of depression. Evidence from other studies also shows that similar strategies are useful for both targeted and universal programmes for the prevention of perinatal depression [26–30]. Moreover, the literature shows that preventive interventions not only reduce the burden of perinatal depression but also contribute to improving recipients’ help-seeking attitude and psychosocial functioning [5, 31].

However, it is also recognised that for an intervention to be successful, it needs to take into consideration the needs of the target population along with their social, religious, and cultural norms [22]. Our adaptation process took into consideration these factors that can impact the well-being of expectant women. For instance, illustrations were re-drawn depicting Turkish women in everyday scenarios, the language was simplified to address non-depressed women, messages were generalised and relatable examples were used. Consequently, the THP-group version was perceived as comprehensible, relatable, and acceptable to its target population. Similar adaptations to the THP in other settings demonstrated its cross-cultural adaptability. For instance, in India and Pakistan, THP was adapted for delivery through the local lay volunteers [8, 22, 32]. In China, THP was adapted to be delivered by nurses and integrated into the existing healthcare system [23]. In Vietnam, it was adapted for universal maternal and child healthcare intervention [22]. Small adaptations similar to those conducted with the Turkish version led to a version acceptable and relevant to the needs of the target population. However, large-scale trials are needed to establish the effectiveness and feasibility for the implementation of these adapted versions.

Our approach to integrate maternal mental health in hospital and community settings is necessary to achieve parity in maternal health care, especially in deprived populations. Integrating the preventive intervention into an existing general health programme delivered by an existing cadre of antenatal nurses can be a potential way forward in Turkey. The THP’s key advantage is that it is designed to be integrated into existing health systems and hence scalable. Adapting the evidence-based THP for use in the group setting and integrating it in antenatal routine sessions can be an effective step in the management of perinatal depression. This model may offer effective mental health prevention without adding to the existing burden on antenatal nurses and also eliminates the need to add another cadre of workers to the existing health system.

The effectiveness of the modified intervention on the mother’s mental health was not examined with an adequate sample due to the study’s limited scope and resource limitations. Field testing did not include extensive process evaluation and was limited to small groups of individuals who were not systematically recruited. Independent research teams could undertake more extensive process evaluations of the adapted version in future studies.

## Conclusion

We conclude that the WHO Thinking Healthy Programme is transferable to the Turkish cultural and healthcare context. Hence the THP-group version has the potential to add value to Turkey’s existing universal healthcare programme by improving the health and well-being of expectant women to bridge the enormous treatment gap for perinatal depression and prevent the serious consequences for women and their children. Further evaluation can help establish the feasibility to scale-up.

## Data Availability

The datasets used and/or analysed during the current study available from the corresponding author on reasonable request.
